# Editorial: Nutrition counseling for non-communicable disease management

**DOI:** 10.3389/fnut.2025.1668677

**Published:** 2025-07-30

**Authors:** Jeanette M. Andrade, Sofi G. Julien

**Affiliations:** ^1^Department of Food Science and Human Nutrition, University of Florida, Gainesville, FL, United States; ^2^Department of Nutrition and Food Sciences, Faculty of Arts and Sciences, Holy Spirit University of Kaslik, Jounieh, Lebanon

**Keywords:** assessment, intervention, recommendations, education, non-communicable diseases, nutrition

## Introduction

Globally, non-communicable diseases (NCDs) like cardiovascular disease, diabetes, cancer, chronic respiratory diseases, infertility, and obesity are the main contributors to mortality and morbidity. The primary modifiable factor influencing the diagnosis and progression of NCDs is often diet. Changing lifestyle habits can be challenging without support; therefore, nutritional counseling by health professionals is essential.

Nutrition counseling refers to the guidance provided by a health professional with specialized training in nutrition to help individuals make healthy food choices and develop sustainable eating habits. Traditionally, nutrition counseling involves a one-on-one approach, where the health professional collects information about knowledge and current habits and then provides tailored recommendations to the individual. Given the limited evidence that the one-on-one counseling approach leads to improved lifestyle behaviors, alternative methods have been explored that include counseling with theories and frameworks, family and social network involvement, and the use of technology. Results from these various approaches showed positive improvements; however, identifying a single approach that is more effective in improving dietary habits than others are challenging.

This Research Topic features 11 articles in which 66 authors from Australia, China, Cyprus, Italy, Iran, Mexico, Singapore, Saudi Arabia, and the United States, addressed aspects of nutrition counseling with various NCD states.

Initially, Worthington et al. illustrated the importance of nutritional trial design to improve dietary behaviors through a small sample of 12 interviews. Based on their findings, the most common techniques to enhance participant compliance were extensive screening protocols, designing the studies with several behavioral-change techniques, approaches from other successful studies, and considering potential participant barriers.

There was an exploration toward healthcare workers in Italy, who were deemed as working in high stress environments, and their risk for NCDs by Pirrello et al.. From the 273 respondents, some did not adhere to a healthy lifestyle and were at risk for a NCD. For example, 33.7% of the respondents indicated that they wanted to increase their intake of fruits and vegetables weekly, yet 27.5% implemented this change. Even though working in a healthcare facility may create an assumption that one adheres to healthy lifestyles, current habits and workload may prevent one from changing and adapting to a lifestyle that is most suitable to reduce NCDs. A study conducted by Alqarni et al. with a total of 1,068 healthcare professionals, found that 58% believed the ketogenic diet could enhance the quality of life for adults with chronic obstructive pulmonary disease (COPD). Specifically, the research emphasized the therapeutic potential of this diet in managing COPD, particularly regarding its anti-inflammatory properties and ability to modify symptoms.

Wang et al. discovered how a body roundness index could help in identifying those at risk for NCDs among 2,319 females from the 2013-2019 NHANES dataset. The large-scale cohort Chinese study conducted by Li et al. explored the relationship between body composition in early pregnancy and the risk of developing gestational diabetes mellitus (GDM), which was shown to be negatively correlated with free-fat mass and lean mass but positively associated with percentage of body fat and fat mass. The findings support the idea that routine prenatal care should include body composition measurements to enable the early identification and prevention of GDM. Qiu et al. explored nutritional status by using CONUT (Controlling Nutritional Status) and NRI (Nutritional Risk Index) among 2,427 individuals on peritoneal dialysis with potential effect on all-cause mortality. At least in this population, 79.1% of individuals were identified at nutritional risk with the NRI whereas 76.6% were identified at nutritional risk with CONUT. Regardless of the instrument, one who had been classified as severe nutritional risk had higher mortality. Each instrument can detect nutritional risk so it can be instrumental as a screening tool. Another study conducted by Ge et al. involving a cohort of 701 critically ill Chinese adults with intestinal obstruction (IO) demonstrated that a higher prognostic nutritional index (PNI)—which indicates their nutritional and immune status—correlates with a lower likelihood of dying during their hospital stay. The findings have significant clinical implications because incorporating the PNI, which is routinely used as a preoperative tool assessment, into the standard risk assessment protocol upon ICU admission enables clinicians to promptly identify patients with inadequate nutritional and immune profiles who are at a higher risk for adverse outcomes. The study conducted by Yu et al. examined the effectiveness of various sarcopenia screening methods among 300 adults with a bone tumor using the Asian Working Group for Sarcopenia screening tool. They demonstrated that the most effective approach for measuring muscle mass in the screening and follow-up of malnutrition involves combining SARC-F, SARC-Calf, and SARC-F+BEM measurements, serving as an alternative to ionizing imaging methods.

Michail et al. developed a questionnaire to assess nutritional knowledge, perceptions, and sustainability for a plant-dominant low-protein diet in adults with chronic kidney disease (CKD). This study points out the need to address culture-based dietary habits to benefit people at risk or with CKD throughout the nutritional care process. Furthermore, a higher dietary quality can reduce the frequency and severity of migraine episodes as identified by the Iranian study conducted by Feyzpour et al.. The authors concluded that prioritizing overall diet quality instead of concentrating solely on individual macronutrients or micronutrients is a promising approach for improving the prognosis and overall condition of individuals suffering from migraines. Finally, a study by Sevilla-González et al. explored how a tailored nutrition care process could be implemented for individuals with metabolic diseases in both inpatient and outpatient settings in Mexico. From the researchers and clinicians who were able to implement this process, it was successful. However, for this to be successfully implemented within other countries and populations, a thorough review and discussion needs to take place.

Overall, these studies involved diverse populations from around the world, allowing for adaptability and testing in other groups. The findings will contribute to developing a more effective consensus on the best approaches to use during nutrition counseling aimed at improving lifestyle habits and ultimately reducing NCDs globally.

